# The doctor will not see you now: investigating the social determinants of specialist care using the Canadian Longitudinal Study on Aging (CLSA)

**DOI:** 10.3389/fpubh.2024.1384604

**Published:** 2024-09-27

**Authors:** Marie Lan, Feben W. Alemu, Shehzad Ali

**Affiliations:** ^1^Department of Epidemiology and Biostatistics, Western University, London, ON, Canada; ^2^Schulich Interfaculty Program in Public Health, Schulich School of Medicine and Dentistry, Western University, London, ON, Canada; ^3^Department of Health Sciences, University of York, Heslington, York, United Kingdom; ^4^WHO Collaborating Centre for Knowledge Translation and Health Technology Assessment in Health Equity, Ottawa, ON, Canada; ^5^Department of Psychology, Macquarie University, Sydney, NSW, Australia

**Keywords:** CLSA, health care access, health care utilization, older adults, medical specialist

## Abstract

**Background:**

The Canada Health Act mandates universal access to medical services for all Canadians. Despite this, there are significant disparities in access based on socioeconomic status, race and ethnicity, immigrant status, and indigeneity. However, there is limited evidence on the use of specialist services among older adults in Canada. The primary objective of this study is to identify the associations of social determinants of health with access to medical specialist services for Canadians aged 45 years and older. The second objective is to identify the reasons for not being able to access the needed specialist care.

**Methods:**

A cross-sectional analysis of the Canadian Longitudinal Study on Aging survey was conducted. Based on the Andersen’s model of health services use, a multivariable logistic regression model was used to evaluate the associations between ‘not being able to access the needed specialist service(s) in the last 12 months’ and individual-level sociodemographic determinants.

**Results:**

Approximately 97% of those who required specialist care in the last year were able to visit a specialist. Of the participants who were not able to access the needed specialist services, about half (50.90%) were still waiting for a visit. The following factors were associated with greater difficulty in accessing specialist care: being younger (45-54 years), living in a rural area, having some post-secondary education, having a household income below $50,000 a year, not having a family physician, and having fair or poor perceived general health. Residents of British Columbia and Nova Scotia had a higher likelihood of reporting difficulty compared to those residing in Ontario.

**Conclusion:**

While a majority of respondents were able to access specialist services when needed, those who had difficulty in accessing care were more likely to come from socially marginalized groups. Targeted policy interventions and improved health system coordination can reduce these barriers to care.

## 1 Introduction

Universal health care access based on need is the founding principle of the Canadian health care system (1). The Canada Health Act mandates universal access to medical services for all Canadians. Despite this, there are significant disparities in access based on socioeconomic status, race and ethnicity, immigrant status, and indigeneity (2). Identifying and minimizing inequities in access is essential to upholding the underlying value of the health system (1).

Andersen’s Behavioral Model of Health Services Use is frequently used to understand the determinants of health care access and use (3). It is a theoretical framework used to understand access to and utilization of health services and acknowledges the factors that impact the decision to use or not use health services (4). The model predicts that a sequence of three categories of determinants: predisposing, enabling, and need-based impact health service utilization. Predisposing factors reflect an individual’s propensity to use health services (5) and include age, sex at birth, and immigration status. Enabling factors include resources that may facilitate or create barriers to accessing health services such as education level and income. Need-based factors reflect an individual’s perception of their own health and their ability to recognize that they require health care. These include perceived health and number of chronic conditions. These factors act at the patient, provider, and community levels of care and impact the accessibility of health care services.

Difficulties in accessing health care services can lead to unmet health care needs which are the difference between services judged to be necessary to appropriately deal with health problems and services individuals receive (6). Unmet needs result from a variety of reasons, including barriers to availability, accessibility (transportation, service hours, wait times, etc.), and acceptability (attitudes toward illness) of health services (7). Most health systems have multiple levels of care which progress from common, low-acuity health conditions to more complex and high-intensity care in hospitals. In most cases, specialist care is accessed through a referral from a primary care provider (8). While determinants of primary care utilization are extensively researched (9–12), there is limited literature on access to specialist care (13).

Among patients seeking specialist care, long waiting times are cited as the most significant barrier to health care services (13). From 2014 to 2016, the national median wait time for all specialist referrals, including urgent cases, was 11 weeks, with a quarter of patients having to wait 25 weeks or longer for an appointment (14). Compared to other high-income countries, Canada ranks among the worst for specialist care wait times with 61% waiting a month or more for a specialist appointment in 2016 (15). In contrast, Germany and Switzerland had 25 and 23% of people waiting more than 1 month, respectively (15). Difficulty getting an appointment and long wait times in the physician’s office have been cited as the second- and third- most common reasons for difficulty accessing specialist care between 2009 and 2013 (16).

There have been some provincial and national surveys evaluating social determinants of having access to specialist care (13, 16). A 2011 survey of Ontario residents found that of those requiring a specialist visit, 22% reported some difficulty getting the care they needed. Newcomers and longer-term immigrants (>10 years) were more likely to experience difficulties with wait times compared to non-immigrants. Those with post-secondary education, those living in urban areas, and those with one or more chronic conditions also experienced more difficulties (13).

The average age in Canada in 2021 was 41.7 years. In a medium-growth projection, the average age is projected to be 45.1 in 2068 (17). The proportion of those aged 65 and older is also projected to increase from 18.5% in 2021 to 25.9% in 2068 (17). Due to the aging population, older adults may require health care services that may not be adequately supported by the current system. Furthermore, middle-aged, and older adults are more likely to require the use of specialist services due to increased multimorbidity (18). The primary objective of this study is to identify associations between social determinants of health and access to the needed medical specialist services in Canadians aged 45 years and older. The second objective is to identify reasons for not being able to access the needed specialist care.

## 2 Methods

The Canadian Longitudinal Study on Aging (CLSA) (19) is a national 20-year prospective cohort study of adults aged 45 to 85 years at the time of recruitment. There were over 51,000 participants from across the 10 provinces in 2011 (20). Participants were recruited into either a Comprehensive or a Tracking cohort. The Comprehensive cohort is comprised of 30,097 participants sampled from the provincial health registration databases, and random digit dialing (21). Data were collected through an in-home interview and a visit to one of eleven data collection sites for physical assessments and biospecimen collection. Participants in the Comprehensive cohort live within a 25-50 km radius of the collection sites, which are located across seven provinces (22). The Tracking cohort is comprised of 21,240 participants who were sampled from the Canadian Community Health Survey, provincial health registration databases and random digit dialing (22) across 10 provinces. Data for the Tracking cohort were collected through telephone interviews (22). There was no overlap between the comprehensive and tracking cohorts. In addition, the sampling methods and core measurement tools were harmonized between Tracking and Comprehensive cohorts (18). Residents of the three territories, persons living on Indigenous reserves or Crown lands, institutionalized persons, full-time members of the Canadian Forces, individuals who are cognitively impaired, individuals who cannot communicate in English or French, and residents of some remote regions were excluded (22). Participants who became institutionalized during the study period were followed either through personal or proxy interviews. The full CLSA protocol has been described previously (22).

The current study is a cross-sectional analysis of the first follow-up wave (2015–2018). Health and health care utilization data such as perceived health, chronic conditions, and specialist care data were collected in the first follow-up. Sociodemographic characteristics such as age, household income, and education were collected at baseline (2011–2015). The survey questions analyzed in this study were identical for both tracking and comprehensive cohorts. This study has received ethics approval from Western University’s Research Ethics Board.

### 2.1 Measurement of access to specialist care

The primary outcome of interest was the use of specialist care services. Participants were asked: “During the past 12 months, have you had contact with any of the following about your physical or mental health?” where one option is “medical specialist (such as a cardiologist, gynecologist, psychiatrist or ophthalmologist).” Respondents who indicated that they did not visit a medical specialist were asked: “Why have you NOT seen a medical specialist (such as a cardiologist, gynecologist, psychiatrist or ophthalmologist) in the past 12-months?” of which options included: not needed, difficulty getting a referral, difficulty getting an appointment, no specialists in the area, transportation problems, language problem, personal and family responsibilities, appointment cancelled or deferred by specialist, still waiting for a visit, unable to leave the house due to health condition, and other. Those who were able to visit a specialist were categorized as not experiencing barriers and those who required care but did not visit a specialist were classified as not being able to access the needed specialist care.

### 2.2 Measurement of social determinants

**Figure 1:**
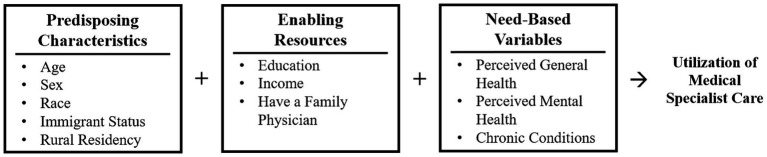
Andersen’s behavioral model of health services use applied to ability to access medical specialist care.

Independent variables were classified into predisposing, enabling, and need-based factors (Figure 1) according to Andersen’s Behavioral Model of Health Services Use (3). Predisposing factors include age, sex at birth, race, immigration status, and place of residence. Age was reported as a continuous variable and categorized into aged 45–54 years old, 55–64 years old, 65–74 years old, and over 75 years old. Sex at birth was a binary variable: male or female. The CLSA uses Statistics Canada’s census classification of racial groups: white only, black only, Korean only, Filipino only, Japanese only, Chinese only, South Asian only, Southeast Asian only, Arab only, West Asian only, Latin American only, other racial or cultural origin (only), and multiple racial or cultural origins. Due to small numbers in the non-white ethnicities, they were grouped together and race was dichotomized into white or non-white. Immigrant status was dichotomous: immigrant or non-immigrant, with immigrants being defined as people born outside of Canada, and non-immigrants being people born in Canada. Place of residence was also dichotomous: urban or rural. “Rural” includes populations living in rural areas within and outside of census metropolitan areas and census agglomerations, in accordance with Statistics Canada Postal Code conversions (23).

Enabling factors include education level, income, and having a family physician. Education was categorized as having less than a secondary school diploma, secondary school diploma, no post-secondary education, and post-secondary education or higher. Total household income was measured in Canadian Dollars and categorized into five categories: <$20,000; $20,000 to $49,999; $50,000 to $99,999; $100,000 to $149,999 and ≥ $150,000. Having a family physician was dichotomous: yes or no.

Need-based variables reflect an individual’s perception of their own health and their ability to recognize that they require health care. These include perceived general health, perceived mental health, and having chronic conditions. Participants were asked to rate their perceived general and mental health as either excellent, very good, good, fair, or poor. Participants were also asked about their chronic conditions which included 45 different conditions. Due to the variability in the included chronic conditions, they were not weighed by type and/or severity. A count variable was created, and participants were categorized as having either zero, one, two, or three or more chronic conditions.

### 2.3 Statistical analysis

Univariable analysis used inflation weights following CLSA survey developer recommendations (24). Mean and standard deviation was computed for continuous variables and proportion for categorical variables. Multivariable logistic regressions were conducted in accordance with Andersen’s Behavioral Model of Health Services Use (3). The dependent variable was “not being able to access the needed specialist services.” Those who visited a specialist were coded as “0” and those who did not access specialist care when needed were coded as “1.” The analysis was conducted in four stages. The first model included only predisposing variables; the second model included predisposing and enabling variables; the third model included predisposing, enabling, and need variables; the final model included predisposing, enabling, and need variables and accounted for differences between the provinces. All regression models used analytic weights, as recommended by CLSA survey developers (24). Variance inflation factors (VIF) were estimated in a linear regression model to assess multicollinearity between the independent variables. Based on Vittinghoff et al. (39), values greater than 10 were considered problematic. The VIFs for all models were under 1.5, indicating an absence of multicollinearity. All statistical analyses were performed using STATA 17.0, StataCorp LLC, College Station, TX.

## 3 Results

### 3.1 Univariable analysis

Complete data was available for 22,145 survey participants: 14,006 from the Comprehensive and 8,139 from the Tracking cohorts. There were 23,873 participants who were excluded because they either did not require specialist care (*n* = 16,249), did not indicate whether they had visited a specialist (*n* = 77), did not indicate a reason for why they did not receive specialist care (*n* = 36), or did not have available data (*n* = 7,511). There were 5,319 participants who were missing data for at least one independent variable, with 3,381 excluded for missing chronic conditions data and 1,743 excluded for missing total household income.

Characteristics of the study sample using inflation weights are shown in Table 1. The average age of the participants was 60.13 years. Female participants made up 52.29% of the sample. The sample was 95.23% white-identifying, 14.82% were immigrants, and 20.71% resided in rural areas. Additionally, 61.90% had a post-secondary diploma or higher and 66.89% had yearly household incomes of less than $100,000. Overall, 94.46% had a family physician. About half of the sample had excellent or very good perceived general health (13.13 and 37.29% respectively), 33.05 had good health, 13.41% had fair health, and 3.12 had perceived health. Most of the sample had excellent or very good perceived mental health (22.63 and 40.96% respectively), 27.95 had good perceived mental health, 7.37 had fair perceived mental health, and 1.10% had poor perceived mental health. About three-quarters of participants had three or more chronic conditions. Ontario and Quebec residents comprised most of the sample (38.45 and 25.08% respectively). Additionally, 97.46% of the sample had visited a specialist within the past 12 months, while 2.54 did not.

**Table 1:** Descriptive statistics for patients who required a visit to a specialist.

Variable	Weighted proportion (%)
*Age (years), (Mean(SD))*	60.13 (10.03)
*Age group (years)*	
45–54	35.79
55–64	32.89
65–74	20.43
75+	10.89
*Sex at birth*	
Male	47.71
Female	52.29
*Race*	
White	95.24
Non-White	4.76
*Immigrant*	
No	85.18
Yes	14.82
*Urban/Rural*	
Urban	79.29
Rural	20.71
*Education*	
Post-secondary diploma or higher	61.90
Some post-secondary	8.96
High school diploma	12.46
Less than high school	16.68
*Household income*	
≥$150,000	14.55
$100,000–$149,999	18.56
$50,000–$99,999	37.14
$20,000–$49,999	24.68
<$20,000	5.07
*Has family physician*	
Yes	94.46
No	5.54
*Perceived physical health*	
Excellent	13.13
Very Good	37.29
Good	33.05
Fair	13.41
Poor	3.12
*Perceived mental health*	
Excellent	22.63
Very Good	40.96
Good	27.95
Fair	7.37
Poor	1.10
*Chronic conditions*	
0	3.23
1	8.51
2	13.39
3+	74.87
*Province*	
Alberta	9.62
British Columbia	14.58
Manitoba	3.28
New Brunswick	2.05
Newfoundland and Labrador	1.64
Nova Scotia	2.49
Ontario	38.45
Prince Edward Island	0.41
Quebec	25.08
Saskatchewan	2.41
*Visit to a specialist in past 12 months*	
Yes	97.46
No	2.54

*N = 22,145.*

**Figure 2:**
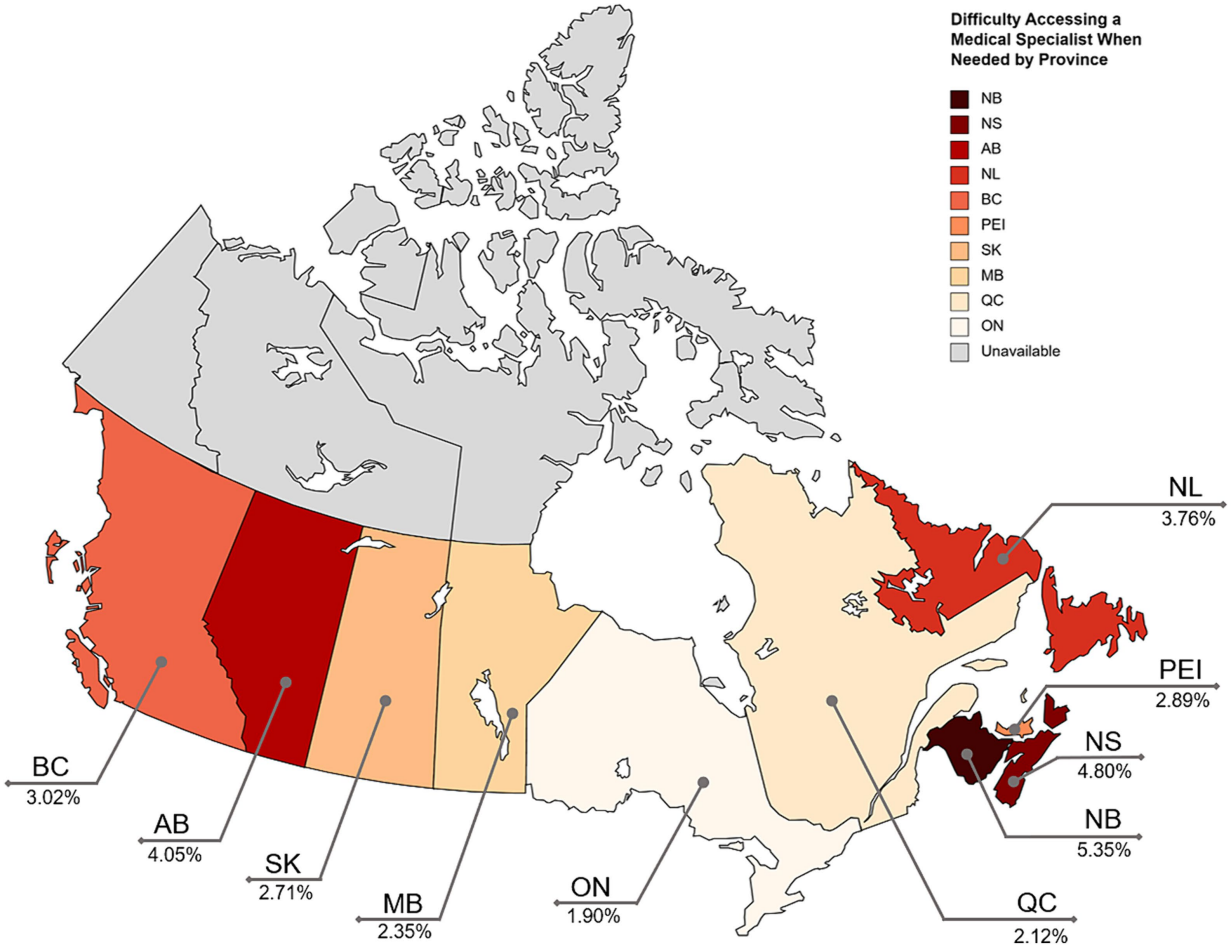
Percentage of individuals in the provinces with reported difficulty accessing a specialist when needed. Darker colouring represents a higher percentage of reported difficulty. Created with mapchart.net.

Use of specialist care services differed across the provinces with residents in New Brunswick (5.35%), Nova Scotia (4.80%), and Alberta (4.05%) reporting the highest proportion of people not being able to access specialist care in the past 12 months (Figure 2). Residents of Ontario (1.90%), Quebec (2.12%), and Manitoba (2.35%) reported the lowest proportions of difficulty. Of the participants who did not visit a specialist when needed in the last 12 months, about half (50.90%) indicated they did not visit because they were still waiting for their appointment (Table 2). Of those who reported difficulty, 18.78% reported having difficulty getting an appointment, 14.78% had difficulty getting a referral, and 17.28% had trouble due to other reasons. Still waiting for an appointment was the highest reported reason for not being able to access the needed specialist care in Alberta (76.71%), British Columbia (55.13%), Manitoba (45.72%), New Brunswick (32.47%), Nova Scotia (66.72%), and Ontario (59.53%). The most reported reason for not accessing needed specialist care was difficulty getting an appointment in Prince Edward Island (67.05%) and Saskatchewan (41.99%), difficulty getting a referral in Quebec (32.61%). In Newfoundland and Labrador, other reasons (42.17%) followed by personal and family responsibilities (27.41%) were the most reported reasons for not accessing needed specialist care.

**Table 2:** Reasons for not accessing needed specialist care by province.

Reason for not accessing needed specialist care	Overall (*n* = 547)	Alberta (*n* = 62)	British Columbia (*n* = 136)	Manitoba (*n* = 36)	New Brunswick (*n* = 23)	Newfoundland and Labrador (*n* = 28)	Nova Scotia (*n* = 63)	Ontario (*n* = 92)	Prince Edward Island (*n* = 12)	Quebec (*n* = 85)	Saskatchewan (*n* = 10)
Still waiting for visit	50.9	76.71	55.13	45.72	32.47	10.98	66.72	59.53	16.14	25.73	29.07
Difficulty getting an appointment	18.73	6.33	9.17	26.96	27.52	10.49	19.19	20.00	67.05	27.91	41.99
Difficulty getting a referral	14.78	4.26	9.01	33.19	2.71	3.07	10.91	12.38	16.96	32.61	14.45
No specialists in the area	4.07	0.00	3.86	4.77	31.90	4.28	8.59	0.00	5.92	6.40	0.00
Personal and family responsibilities	3.74	0.00	4.09	3.77	0.00	27.41	0.00	7.30	0.00	0.73	0.00
Appointment cancelled or deferred by specialist/doctor	2.45	1.12	0.41	0.85	0.00	1.87	0.53	1.80	0.00	5.33	18.65
Language problem	1.20	0.00	0.00	0.00	0.00	0.00	0.00	0.00	0.00	5.70	0.00
Transportation problems	0.77	0.00	2.88	1.16	0.00	4.28	0.00	0.00	4.52	0.51	0.00
Unable to leave the house due to health condition	0.61	0.00	3.41	0.00	0.00	0.00	0.00	0.00	4.52	0.00	0.00
Other	17.28	14.31	19.23	9.77	8.29	42.17	12.28	15.89	41.37	20.58	15.89

### 3.2 Regression analysis

The results of the regression analyses are presented in Table 3. For the model consisting of predisposing variables, participants aged 65–74 years (OR = 0.70, 95% CI: 0.43, 0.86) and 75 years and older (OR = 0.41, 95% CI: 0.27, 0.63) had lower odds of not being able to access the needed specialist care compared to those aged 45–54 years. Those who lived in rural areas had 1.53 times higher odds of not accessing needed specialist care compared to those living in urban areas (OR = 1.53, 95% CI: 1.12, 2.09).

**Table 3:** Logistic regression for having difficulties receiving medical specialist care.

	Predisposing OR (95% CI)	*p*-value	Predisposing + Enabling OR (95% CI)	*p*-value	Predisposing + Enabling + Need OR (95% CI)	*p*-value	Predisposing + Enabling + Need + Province OR (95% CI)	*p*-value
*Age*								
45–54	Ref		Ref		Ref		Ref	
55–64	0.90 (0.68, 1.21)	0.496	0.83 (0.61, 1.13)	0.229	0.87 (0.64, 1.19)	0.384	0.87 (0.63, 1.19)	0.377
65–74	0.61 (0.43, 0.86)	0.005	0.49 (0.33, 0.74)	0.001	0.55 (0.36, 0.82)	0.004	0.54 (0.36, 0.82)	0.003
75+	0.41 (0.27, 0.63)	<0.001	0.30 (0.18, 0.50)	<0.001	0.33 (0.19, 0.55)	<0.001	0.32 (0.19, 0.54)	<0.001
*Sex at birth*								
Male	Ref		Ref		Ref		Ref	
Female	0.97 (0.75, 1.25)	0.830	0.94 (0.73, 1.20)	0.601	0.96 (0.74, 1.25)	0.770	0.96 (0.74, 1.24)	0.741
*Race*								
White	Ref		Ref		Ref		Ref	
Non-White	1.06 (0.53, 2.11)	0.872	0.98 (0.50, 1.90)	0.948	0.94 (0.50, 1.78)	0.852	0.96 (0.52, 1.75)	0.889
*Immigrant*								
Non-Immigrant	Ref		Ref		Ref		Ref	
Immigrant	0.86 (0.57, 1.30)	0.470	0.92 (0.60, 1.39)	0.628	0.90 (0.60, 1.37)	0.629	0.90 (0.59, 1.37)	0.625
*Urban/Rural*								
Urban	Ref		Ref		Ref		Ref	
Rural	1.53 (1.12, 2.09)	0.007	1.46 (1.06, 2.02)	0.021	1.47 (1.06, 2.02)	0.020	1.48 (1.06, 2.05)	0.021
*Education*								
Post-secondary diploma or higher			Ref		Ref		Ref	
Some post-secondary			1.47 (1.03, 2.09)	0.034	1.45 (1.02, 2.06)	0.039	1.44 (1.01, 2.05)	0.043
High school diploma			1.03 (0.71, 1.48)	0.889	1.01 (0.70, 1.45)	0.966	1.03 (0.72, 1.48)	0.871
Less than high school			1.61 (0.95, 2.71)	0.077	1.55 (0.92, 2.61)	0.102	1.62 (0.97, 2.71)	0.066
*Total household income*								
>$150,000			Ref		Ref		Ref	
$100,000–$149,999			1.52 (1.04, 2.23)	0.030	1.44 (0.99, 2.09)	0.056	1.44 (0.98, 2.11)	0.061
$50,000–$99,999			1.450 (0.99, 2.11)	0.054	1.32 (0.90, 1.92)	0.153	1.34 (0.89, 2.01)	0.157
$20,000–$49,999			1.99 (1.33, 2.99)	0.001	1.73 (1.15, 2.60)	0.008	1.79 (1.18, 2.73)	0.006
<$20,000			2.41 (1.36, 4.27)	0.003	1.94 (1.09, 3.44)	0.024	1.98 (1.10, 3.56)	0.023
*Have family physician*								
Yes			Ref		Ref		Ref	
No			2.03 (1.34, 3.08)	0.001	2.10 (1.39, 3.17)	<0.001	2.24 (1.48, 3.40)	<0.001
*Perceived general health*								
Excellent					Ref		Ref	
Very Good					1.32 (0.88, 2.00)	0.184	1.34 (0.89, 2.03)	0.166
Good					1.49 (0.95, 2.34)	0.079	1.51 (0.96, 2.37)	0.076
Fair					2.06 (1.21, 3.52)	0.008	2.09 (1.23, 3.57)	0.007
Poor					2.35 (1.11, 4.96)	0.026	2.23 (1.08, 4.64)	0.031
*Perceived mental health*								
Excellent					Ref		Ref	
Very Good					1.22 (0.85, 1.74)	0.275	1.18 (0.83, 1.68)	0.348
Good					1.28 (0.87, 1.88)	0.209	1.23 (0.84, 1.81)	0.292
Fair					1.34 (0.81, 2.22)	0.253	1.27 (0.77, 2.10)	0.255
Poor					1.39 (0.53, 3.66)	0.509	1.35 (0.51, 3.58)	0.547
*Chronic conditions*								
0					Ref		Ref	
1					0.93 (0.48, 1.81)	0.841	0.93 (0.48, 1.81)	0.821
2					1.18 (0.62, 2.26)	0.613	1.19 (0.62, 2.29)	0.602
3+					0.86 (0.48, 1.52)	0.594	0.87 (0.49, 1.57)	0.651
*Province*								
Ontario							Ref	
Alberta							1.70 (0.95, 3.04)	0.072
British Columbia							1.65 (1.14, 2.38)	0.008
Manitoba							1.12 (0.61, 2.04)	0.714
New Brunswick							1.89 (0.95, 3.74)	0.070
Newfoundland and Labrador							1.00 (0.53, 1.88)	0.995
Nova Scotia							2.13 (1.36, 3.33)	0.001
Prince Edward Island							1.07 (0.47, 2.43)	0.879
Quebec							1.02 (0.67, 1.57)	0.915
Saskatchewan							1.25 (0.56, 2.81)	0.583

*N = 22,145.*

OR, Odds Ratio; CI, Confidence Interval; Ref, Reference Group.

In the model consisting of predisposing and enabling variables, those with some post-secondary education had 1.47 times higher odds of reporting difficulty accessing specialist care in the last 12 months compared to those who only had a post-secondary diploma or higher (OR = 1.47, 95% CI: 1.03, 2.09). Compared to participants with total household income over $150,000, lower total household income was generally associated with higher odds of not being able to access the specialist care ($100,000–$149,999: OR = 1.52, 95% CI: 1.04, 2.23; $20,000–$49,999: OR = 1.99, 95% CI: 1.33, 2.99; <$20,000: OR = 2.41, 95% CI: 1.36, 4.27). In addition, those who did not have a family physician had two times higher odds of not accessing the needed specialist services compared to those who do (OR = 2.03, 95% CI: 1.34, 3.08). Older age continued to be associated with higher access to specialist care and those who live in rural areas continued to have a greater likelihood of not accessing the needed specialist services compared to those who live in urban areas.

For the model consisting of predisposing, enabling and need variables, those who had fair or poor perceived general health had two times higher odds of reporting difficulty accessing specialist services in the last 12 months than those who had excellent perceived health (Fair: OR = 2.06, 95% CI: 1.21, 3.52; Poor: OR = 2.35, 95% CI: 1.21, 3.52). Older age continued to be associated with a decreased chance of not accessing the needed specialist care. Living in a rural area, having some post-secondary education, and not having a family physician also continued to be associated with increased difficulty. Compared to those with total household incomes over $150,000, those with incomes below $50,000 were associated with increased difficulty utilizing specialist services ($20,000–$49,999: OR = 1.73, 95%CI: 1.15, 2.60; <$20,000: OR = 1.94, 95%CI: 1.09, 3.44).

Ontario residents had the lowest rate of difficulty accessing specialist services, so it was chosen as the reference. For the model consisting of predisposing, enabling, need, and Province variables, residents of British Columbia had 1.65 times higher odds of not accessing needed specialist care compared to Ontario residents (OR = 1.65, 95% CI: 1.14, 2.38), while residents of Nova Scotia had 2.13 times higher odds (OR = 2.13, 95% CI: 1.36, 3.33). Adding the provinces to the model did not change any of the previously statistically significant associations from the third model. Sex at birth, race, immigration status, perceived mental health and number of chronic conditions did not have any statistically significant associations with reporting difficulty receiving specialist care in any of the regression models.

## 4 Discussion

This study aimed to identify the associations between social determinants of health and reporting difficulty accessing medical specialist services in the last 12 months among Canadians aged 45 years and older from the 10 provinces. Of the participants who required specialist care, 2.54% were not able to visit a specialist within the past 12 months. This reported difficulty rate is lower than previous projections. A 2016 Statistics Canada report found that 22% of Canadians 15 and older reported difficulty receiving specialized health services (16), however, the study population was limited to those who had received health care that year. A 2011 survey of Ontario residents found that 29% of those surveyed reported difficulty accessing services, but the estimate included those who accessed specialist services (13). The present study found that 1.90% of older Ontario residents were not able to access the needed specialist care in the past 12 months. In the CLSA survey, only those who did not visit a specialist within the last year were asked about the difficulties they experienced. Therefore, the reported difficulty rate applies only to individuals who were not able to see a specialist in the last year, which may underestimate the true rate of difficulty accessing services for the general older population. Furthermore, the present sample consisted of older adults only who are more likely to have a family physician (25), which was found to be associated with decreased difficulty in accessing specialist care.

Of those who did not access the needed specialist services in the last 12 months, “still waiting for a visit” and “difficulty getting an appointment” were the most reported barriers, which is congruent with previous reports of the general Canadian population (13). A survey of Canadian older adults in 2017 found that 59% of older adults reported waiting at least 4 weeks to see a specialist, with 28% waiting 2 months or longer (26). These results call for an increased number of specialists and improved coordination of health care services, and improved inequalities in the referral system.

For the predisposing characteristics, older age was associated with lower odds of not being able to access specialist care which aligns with some previous literature (13, 16), although other research suggests that older adults face more barriers to care (27). It is possible that older adults need care from multiple specialists and are encountering barriers not captured by the survey. Those who visited any specialist within the last 12 months were not asked if they had difficulties. Rural residence was also associated with less utilization of specialist care, supporting the existing literature on the inadequate access rural populations face (28). This calls for the improvement of health care support for those living in rural areas.

The present study did not find that immigration status was associated with access barriers to care in the older population. However, this may be a consequence of the low proportion of immigrants surveyed. Previous literature has evaluated the additional barriers to care immigrants face. Both newcomers and long-term (≥10 years) immigrants have reported greater difficulties accessing specialist care compared to those born in Canada (13). Additionally, a higher proportion of newcomers report experiencing difficulties associated with transportation, cost, and language when accessing specialist care in Canada compared to non-immigrants (13).

Participants with some post-secondary education were more likely to experience barriers to care. Previous literature has also found that individuals with more education were more likely to experience barriers to specialist care (13, 16). This may be a consequence of those with lower education having a lower level of health literacy, leading to less health care-seeking behavior (29). Lower household income was associated with more difficulty accessing specialist care. Although lower income has been associated with increased barriers to care in other countries, previous studies on access to specialist care have not shown inequalities among income groups in Canada (13). The results of the present study show that Canada’s universal health care system may be inadequate in reducing income-based inequalities regarding medical specialist care.

In the present study, individuals without a family physician were found to have significantly more difficulty accessing specialist services. Due to Canada’s two-tiered system, family physicians act as a gatekeeper to many specialty services (30). Because of this, family physicians can take a central role in improving the optimization of specialist services. Pooled referral systems are centralized referral processes that allow family doctors to choose whether to refer patients to the next available specialist or a specific practitioner. Ontario has implemented a pooled referral system for hip and knee replacements (31), and Quebec implemented a general pooled referral system covering 26 specialties (32). Similar programs have been carried out in Newfoundland and Labrador, Saskatchewan, British Columbia, and Alberta for specific specialties (33). Pooled referral systems have been implemented successfully and have reduced wait times in Canada and the United Kingdom (33). Implementing pooled referral systems universally, as described above, could help reduce wait times and improve specialist care access to all Canadians. In addition, access to family medicine services is also impacted by social factors. Women, individuals below the age of 65, and those with lower education have reported more difficulties accessing primary care compared to their counterparts (34). Addressing the social determinants of having a family physician can in turn reduce inequities in accessing specialist care. Participants with worse perceived general health were more likely to experience difficulties in accessing specialist care which is congruent with previous data (16). However, an increased number of chronic conditions was not associated with having greater difficulty. This may be due to the variety of included chronic conditions, some of which may not need consistent monitoring by a specialist (ie. allergies).

There were also some differences between the provinces, with British Columbia and Nova Scotia residents having lower odds of accessing specialist care compared to those in Ontario. The disparity between provinces may be a result of the decentralization of Canadian health care as services are governed provincially (35).

### 4.1 Limitations

The associations examined in this study are limited by the variables available in the CLSA dataset. The survey only included participants from the provinces. There was also an over-representation of those who were white, non-immigrants, and those in the urban areas. In addition, the study population consisted of higher proportions of those who had a post-secondary degree or higher and those with a household income of over $100,000 compared to the general population. Due to these differences, caution should be taken when generalizing the results to specific population groups. Immigrants were not asked about how long they were a resident of Canada, which may impact the associations. In addition, chronic conditions were not weighted based on severity or expected health care use which may have impacted associations as well.

Those who were able to visit a specialist in the last year were not asked about the difficulties they faced to access those services. It is possible that patients were referred to multiple specialists over the last year and that these individuals are experiencing difficulties receiving some specialist services and not others. There was no information on the type of specialist services required or used, or the number of specialists a participant had referrals or connections to (36, 37). Multimorbidity is increasingly present among older Canadians and requires a complex connection of different specialized teams (38). Since this information was not collected, the rate of difficulty experienced may be an underrepresentation of the true reported difficulty using specialist care services. Furthermore, there was no information on how long those who are still waiting for an appointment have been waiting. It is possible that some of the participants are still under the target wait times for the particular service they need. Nevertheless, when those who were still waiting for services were removed, the all associations remained robust.

## 5 Conclusion

The present study identified the determinants of not being able to access the medical specialist care services, when needed, in Canadian adults aged 45 years and older in Canada. Although Canada’s health care system intends to be universal, inequalities between social groups continue to persist. While a majority of respondents were able to utilize specialist services when needed, there are specific populations where there is unmet need. Being younger, living in a rural area, having some post-secondary education, having a lower household income, not having a family physician, and having poorer perceived general health were associated with having increased difficulty accessing specialist care. Those living in British Columbia and Nova Scotia were also found to have increased access to specialist services compared to those in Ontario. The most cited reasons for difficulty receiving specialist care include still waiting for an appointment and difficulty accessing an appointment. Future studies may investigate the barriers to care for specific specialties or procedures to identify areas for improvement.

## Data Availability

The data analyzed in this study is subject to the following licenses/restrictions: data are available from the Canadian Longitudinal Study on Aging (www.clsa-elcv.ca) for researchers who meet the criteria for access to de-identified CLSA data. Requests to access these datasets should be directed to www.clsa-elcv.ca.
